# Effect of Zebularine in Comparison to Trichostatin A on the Intrinsic and Extrinsic Apoptotic Pathway, Cell Viability, and Apoptosis in Hepatocellular Carcinoma SK-Hep 1, Human Colorectal Cancer SW620, and Human Pancreatic Cancer PaCa-44 Cell Lines

**DOI:** 10.22037/ijpr.2021.115097.15196

**Published:** 2021

**Authors:** Masumeh Sanaei, Fraidoon Kavoosi

**Affiliations:** *Research Center for Non-communicable Diseases, Jahrom University of Medical Sciences, Jahrom, Iran.*

**Keywords:** Zebularine, Trichostatin A, Extrinsic, Intrinsic, Pathway

## Abstract

Aberrant histone modifications or promoter region hypermethylation of tumor suppressor genes (TSGs) have been recognized as the important epigenetic molecular mechanism in cancer induction. The potential anticancer activities of histone deacetylase inhibitors (HDACIs) and DNA methyltransferase inhibitors (DNMTIs) have been investigated in recent years. The current study was assigned to investigate the effect of trichostatin A (HDACI) in comparison to zebularine (DNMTI) on the intrinsic pro-apoptotic (*Bax, Bim, and Bak*) and anti-apoptotic (*Bcl-2, Mcl-1, and Bcl-xL*) genes and extrinsic (*DR4, DR5, FAS, FAS-L, and TRAIL* genes) pathways, DNA methyltransferase 1, 3a, and 3b, histone deacetylase inhibitors 1, 2, and 3, cell viability, and apoptosis in hepatocellular carcinoma (HCC) SK-Hep 1, colorectal cancer SW620, and pancreatic cancer PaCa-44 cell lines. The SK-Hep 1, SW620, and PaCa-44 cells were cultured and treated with TSA and zebularine. To determine cell apoptosis, cell viability, and the relative gene expression level, flow cytometry assay, MTT assay, and qRT-PCR were done respectively. The result indicated that zebularine and TSA changed the expression level of the *Bax, Bak, Bim Bcl-2, Bcl-xL, Mcl-1, DR4, DR5, FAS, FAS-L, TRAIL*, *DNA methyltransferase 1, 3a, and 3b, histone deacetylase inhibitors 1, 2, and 3* by which induced cell apoptosis and inhibit cell growth in all three cell lines. Concluding, TSA induced its role through both extrinsic and intrinsic apoptotic pathways in three cell lines, whereas, zebularine played its role via both pathways in the SK-Hep 1cell line, it had no significant effect on *Bcl-2, Bcl-xL, and Mcl-1 *gene expression in SW620 and PaCa-44 cell lines.

## Introduction

Aberrant histone modifications or promoter region hypermethylation of tumor suppressor genes (TSGs) have been recognized as the important epigenetic molecular mechanisms in cancer induction and progression. These mechanisms can inactivate TSGs and cancer-related genes lead to cancer induction and progression. The N-terminal domains of all core histones are subject to diverse modifications, such as methylation, acetylation, phosphorylation, and ubiquitylation at certain residues (1). Acetylation is one of the most prominent posttranslational modifications affecting gene transcription and expression. Histone deacetylases (HDACs) and histone acetyltransferases (HATs) are responsible for the deacetylation and the acetylation of lysines of histone proteins respectively (2). The activity of HDACs condenses the chromatin structure of TSGs resulting in gene silencing and tumorigenesis. Previous analyses have divided HDACs into Class I HDACs (HDAC1, 2, 3, and 8), Class IIa HDACs (HDAC4, 5, 7, and 9), and the class III family of HDACs are also known as Sirtuins (3). 

In addition to histone modification, DNA methylation, primarily at CpG dinucleotides, has long been identified to play an important role in controlling gene expression. Histone modifications and DNA methylation play a significant role in modulating chromatin structure which can control gene expression. DNA methyltransferases (DNMTs), are responsible for DNA methylation, the activity of these enzymes affects chromatin structure and gene expression (4). According to the structure and functions, DNMTs are divided into Dnmt1, Dnmt3a, Dnmt3b, and Dnmt3L (5). The potential anticancer activities of DNA methyltransferase inhibitors (DNMTIs) and HDAC inhibitors (HDACIs) have been investigated in recent years.HDACIs inhibit the activities of HDACs, resulting in an increase in the acetylation of the histone and an enhancement of the gene expression specially TSGs. DNMTIs are widely studied because DNA demethylation re-activates and up-regulates the expression of TSGs that are silenced by DNA hypermethylation. The combination of both HDACI and DNMTI has gained increasing interest as a possible targeted therapeutic cancer therapy (6). 

In-vitro studies have demonstrated that DNMTIs (such as zebularine) and HDACIs (e.g. trichostatin A, TSA) can induce apoptosis through two molecular mechanisms comprising extrinsic and intrinsic apoptotic pathways (7, 8). The intrinsic pathway is controlled by the members of the *BCL-2* family divided into three groups: promoters of apoptosis (*BAX, BAK, and BOK*); inhibitors of apoptosis (*BCL-XL, BCL-2, MCL1, BCL-B, BCL-W, and A1*); and regulatory BH3-only proteins (*BIM, BAD, BID, BIK, BMF, NOXA, HrK, and PUMA*) (9-11). The extrinsic pathway is triggered by the binding of death ligands to their death receptors (DRs) such as TRAIL-R1/DR4, TRAIL-R2/DR5, TRAIL-R3/DcR1, and TRAIL-R4/DcR2 (12). The current study was assigned to investigate the effect of TSA in comparison to zebularine on mitochondrial/intrinsic pro-apoptotic (*Bax, Bim, and Bak*) and anti-apoptotic (*Bcl-2, Mcl-1, and Bcl-xL*) genes and cytoplasmic/extrinsic (*DR4, DR5, FAS, FAS-L, and TRAIL *genes) pathways, *DNA methyltransferase 1, 3a, and 3b, histone deacetylase inhibitors 1, 2, and 3*, cell viability, and apoptosis in hepatocellular carcinoma SK-Hep 1, human colorectal cancer SW620, and human pancreatic cancer PaCa-44 cell lines.

## Experimental

*Materials*

Hepatocellular carcinoma SK-Hep 1, human colorectal cancer cell lines SW620, and human pancreatic cancer PaCa-44 cell lines were purchased from the National Cell Bank of Iran-Pasteur Institute. The zebularine, TSA, and Dulbecco’s modified Eagle’s medium (DMEM) were obtained from Sigma (St. Louis, MO, USA) and dissolved in dimethyl sulfoxide (DMSO) to make a work stock solution. Further concentrations of the compounds, zebularine, and TSA were obtained by diluting the provided stock solution. Other necessary compounds comprising materials and various kits were purchased as provided for previous works (13, 14). The SK-Hep 1, SW620, and PaCa-44 cells were maintained in DMEM supplemented with fetal bovine serum 10% and antibiotics in a humidified atmosphere of 5% CO2 in air at 37 ℃. This work was approved by the Ethics Committee of Jahrom University of Medical science with a code number of IR.JUMS.REC.1399.079.

*Cell culture and cell viability*

 The SK-Hep 1, SW620, and PaCa-44 cells were cultured in DMEM containing 10% FBS (Sigma) and 1% antibiotics which include 10,000 unit/mL penicillin G sodium(Sigma), 10,000 ug/mL streptomycin sulfate, and 25 ug/mL amphotericin B (Sigma) at 37 °C in 5% CO2 °C for 24 h and then seeded into 96-well plates (3 × 105 cells per well). After 24 h, the medium was replaced with an experimental medium containing various concentrations of zebularine (0, 10, 25, 50, 75, 100, μM), and TSA (0, 0.5, 1, 2.5, 5, and 10 μM). The control groups were treated with DMSO, at a concentration of 0.05 %. After 24 h, the SK-Hep 1, SW620, and PaCa-44 cells, experimental and control, were investigated by MTT assay according to Standard protocols to determine cell viability. In this process, the MTT solution was added to each well for 4 h at 37 ℃ and then the MTT solution was changed by DMSO and shaken for 10 min to dissolve all of the crystals. Finally, the optical density was detected at a wavelength of 570 nM by a microplate reader. Each experiment was repeated three times (triplicates). IC50 was analyzed by GraphPad Prism Version 8.0. software. Non-linear regressions of log (inhibitor) vs response were selected for IC50 estimation. The bottom and top parameters were constrained to 0% and 100%.

*Cell apoptosis assay*

To determine SK-Hep 1, SW620, and PaCa-44 cells apoptosis, the cells were cultured at a density of 3 × 105 cells/well and incubated overnight and then treated with zebularine and TSA, based on IC50 values demonstrated in Table 1, for 24 h. Subsequently, the cells were harvested by trypsinization, washed with cold PBS, and resuspended in Binding buffer (1x). Finally, Annexin-V-(FITC) and PI were used according to the protocol to determine the apoptotic cells by FACScan flow cytometry (Becton Dickinson, Heidelberg, Germany).

*Real-time Quantitative Reverse Transcription Polymerase*

*Chain Reaction (qRT-PCR)*

To determine the relative expression level of the *Bax, Bak, Bim, Bcl-2, Bcl-xL, Mcl-1, DR4, DR5, FAS, FAS-L, TRAIL *genes, DNA methyltransferase 1, 3a, and 3b, histone deac-etylase inhibitors 1, 2, and 3 qRT-PCR was done. The SK-Hep 1, SW620, and PaCa-44 cells were treated with zebularine and TSA, based on IC50 values demonstrated in Table 1, for 24 h, except control groups which were treated with DMSO only. Then qRT-PCR was done as our previous works (13, 14). The primer sequences are shown in Table 2 (15-26).

## Results

*Result of cell Cell viability by the MTT assay*

**Figure 1:**
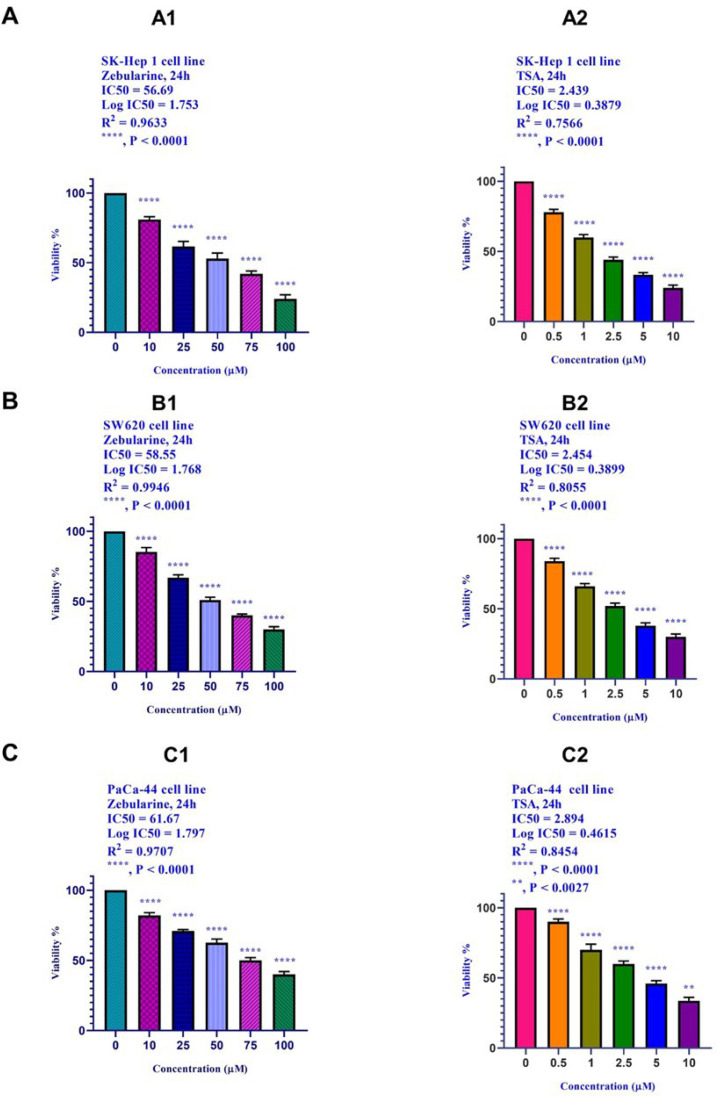
The effect of zebularine (0, 10, 25, 50, 75, 100, μM) and TSA (0, 0.5, 1, 2.5, 5, and 10 μM) on the viability of (A) hepatocellular carcinoma SK-Hep 1, (B) human colorectal cancer cell lines SW620, and (C) human pancreatic cancer PaCa-44 cell line. The cells were treated without and with different doses of zebularine and TSA for 24 and the cell viability was evaluated by MTT assay. Each experiment was achieved in triplicate. Mean values from the three experiments ± standard error of mean are indicated. Asterisks indicate significant differences between treated and untreated cells. ^**^P < 0.0027, and ^****^P < 0.0001

The cell viability of the SK-Hep 1, SW620, and PaCa-44 cells treated with zebularine and TSA was investigated by MTT assay by which the activity of cellular enzymes reduced the tetrazolium salt MTT and produced a dark-blue formazan crystal. The crystal was dissolvable in DMSO to determine the viable cells. As shown in Figure 1, zebularine and TSA induced significant cell growth inhibition ^(****^, *P* < 0.0001 and ^(**^, *P* < 0.0027). IC50 values are shown in Table 1.

*Result of cell apoptosis assay*

**Figure 2:**
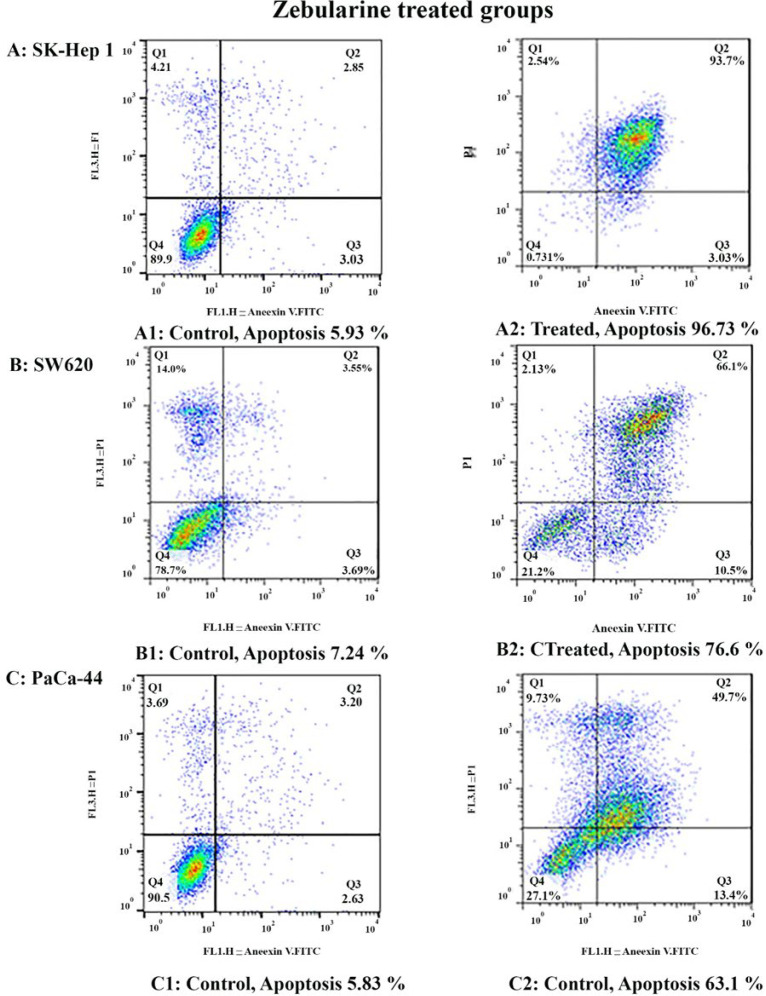
The apoptosis-inducing effect of zebularine was investigated by flow cytometric analysis of (A) SK-Hep 1, (B) SW620, and (C) PaCa-44 cells stained with Annexin V and propidium iodide. The result indicated that zebularine induced cell apoptosis after 24 h of treatment significantly

**Figure 3:**
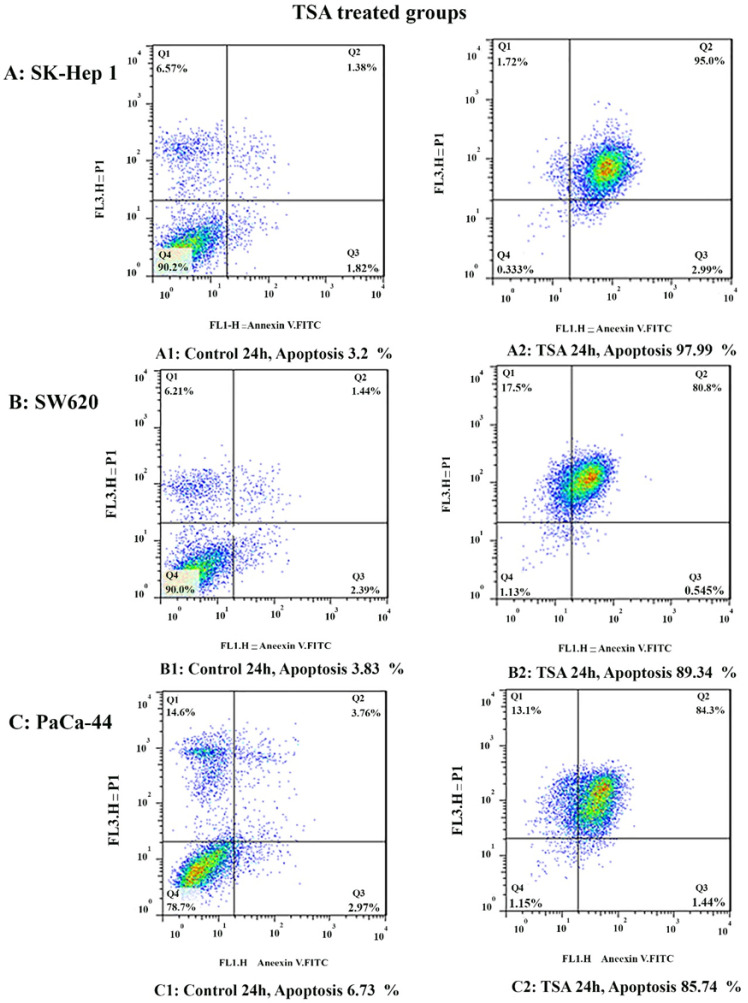
The apoptosis-inducing effect of TSA was investigated by flow cytometric analysis of (A) SK-Hep 1, (B) SW620, and (C) PaCa-44 cells stained with Annexin V and propidium iodide. The result indicated that TSA induced cell apoptosis after 24 h of treatment significantly

**Figure 4:**
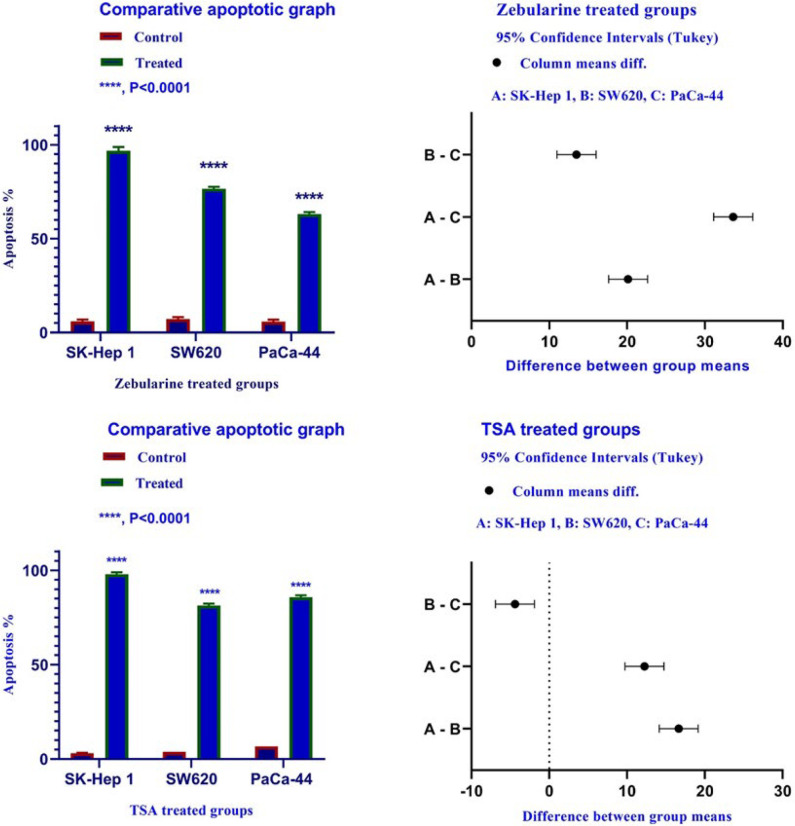
The Apoptotic Effect of zebularine and TSA on SK-Hep 1, SW620, and PaCa-44 cells versus control groups at 24 h. Results were obtained from three independent experiments and were expressed as mean ± standard error of the mean. The results of the statistical analysis indicate significant differences between treated and untreated cells as shown on the right side. ^****^P < 0.0001

To determine apoptosis, the SK-Hep 1, SW620, and PaCa-44 cells were treated with zebularine and TSA, based on IC50 values, for 24 h and then stained using annexin-V-(FITC) and PI to determine apoptotic cells in the early and late apoptosis stage. As indicated in Figures 2–4, both compounds induced cell apoptosis significantly (*P* < 0.001). Maximal and minimal apoptosis was seen in SK-Hep 1 cell treated with TSA and PaCa-44 cell treated with zebularine after 24 h of treatment respectively, Table 3.

*Determination of genes expression*

*Effect of zebularine on the gene expression*

**Figure 5:**
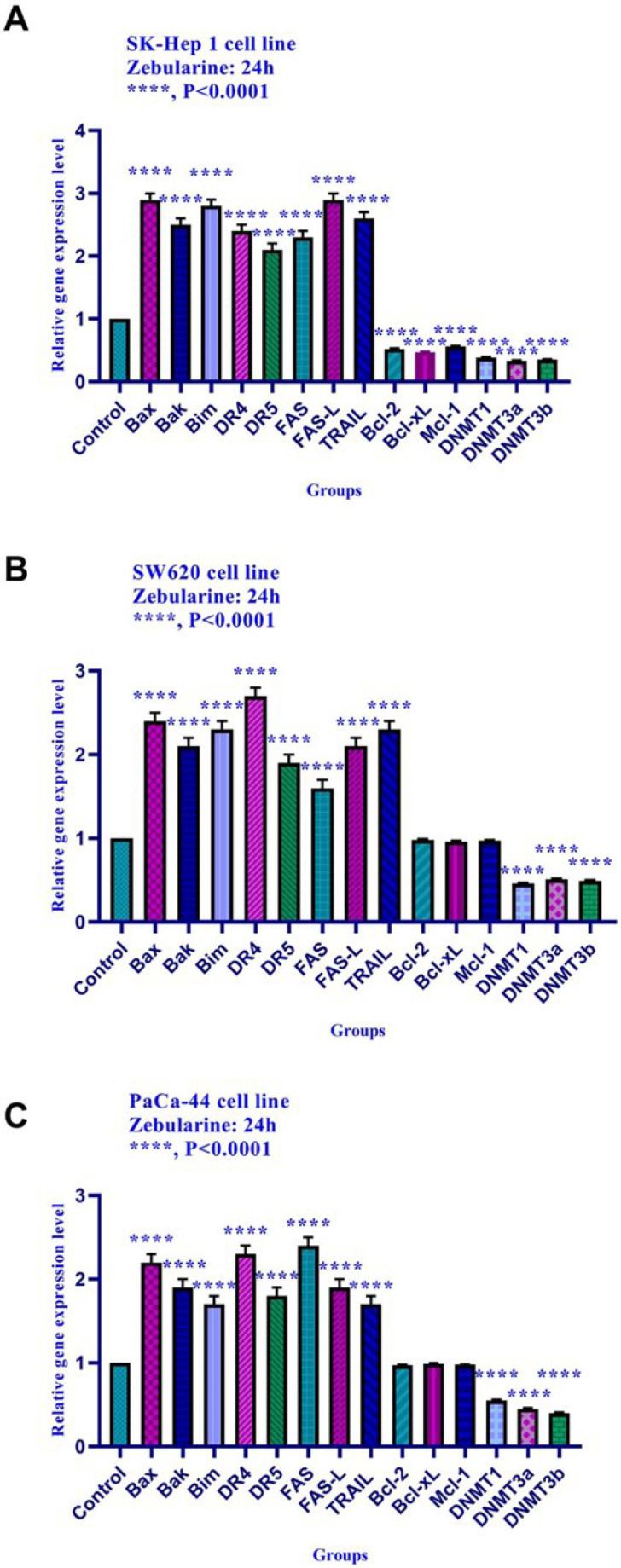
The relative expression level of *Bim, Bax, Bak, Bcl-xL, Bcl-2, Mcl-1, DR4, DR5, FAS, FAS-L, TRAIL, DNA methyltransferase 1, 3a, and 3b, and histone deacetylase inhibitors 1, 2, and 3* in SK-Hep 1, SW620, and PaCa-44 cells treated with zebularine at 24 h. Quantitative reverse transcription-polymerase chain reaction analysis demonstrated that this compound up-regulated the expression of *Bax, Bak, Bim, DR4, DR5, FAS, FAS-L, TRAIL*, and down-regulated the expression of *Bcl-2, Mcl-1, Bcl-xL, DNA methyltransferase 1, 3a, and 3b* significantly. This compound had no significant effect on *Bcl-2, Mcl-1,* and *Bcl-xL*gene expression in SW620, and PaCa-44 cell lines. Asterisks indicate significant differences between treated cells and the control groups. Data are presented as means ± standard error of the mean. ^****^P < 0.0001

The effect of zebularine on gene expression was evaluated by quantitative real-time RT-PCR analysis. The result indicated that zebularine up-regulated the expression of *Bax, Bak, Bim, DR4, DR5, FAS, FAS-L, and TRAIL *genes and down-regulated the expression of *Bcl-2, Mcl-1, Bcl-xL, DNA methyltransferase 1, 3a, and 3b, histone deacetylase 1, 2, and 3* in SK-Hep 1cell line significantly. Additionally, zebularine up-regulated the expression of *Bak, Bax, Bim, DR4, DR5, FAS, FAS-L, and TRAIL *genes and down-regulated the expression of *DNA methyltransferase 1, 3a, and 3b* in SW620, and PaCa-44 cell lines significantly. It had no significant effect on *Bcl-2, Bcl-xL, and Mcl-1* gene expression in SW620, and PaCa-44 cell lines (Figure 5). 

*Effect of TSA on the gene expression*

**Figure 6:**
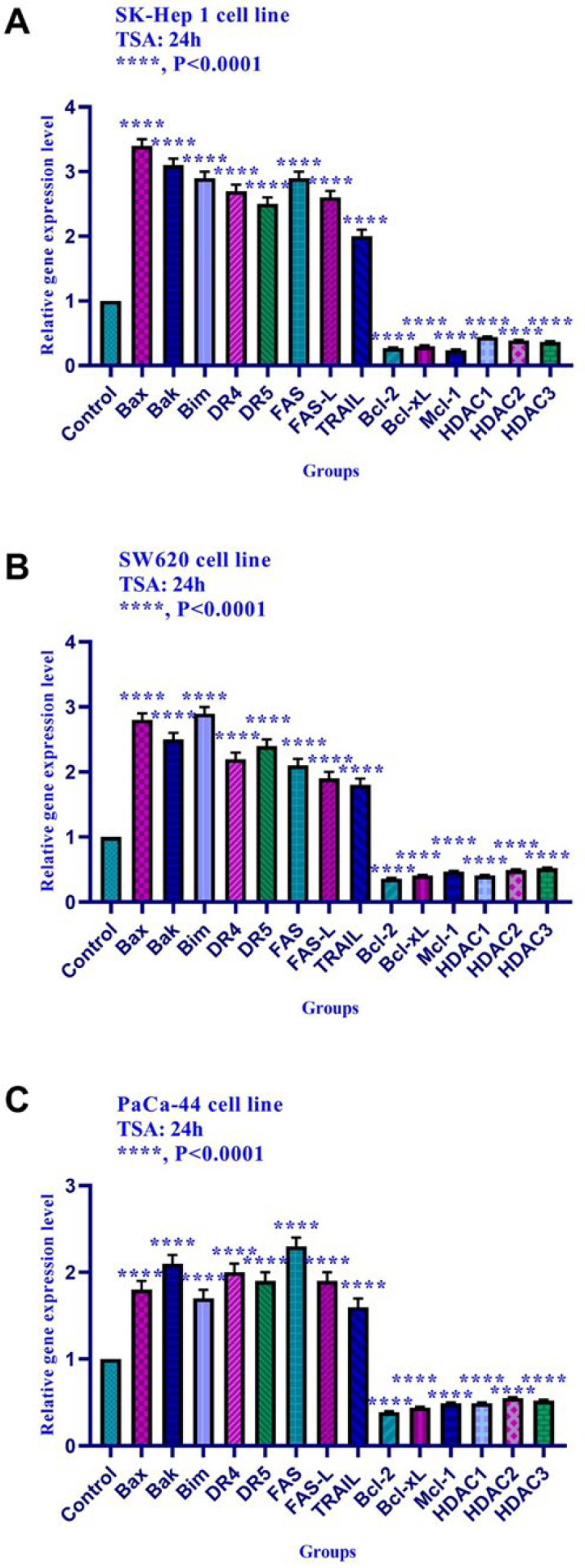
The relative expression level of *Bim, Bax, Bak, Bcl-xL, Bcl-2, Mcl-1, DR4, DR5, FAS, FAS-L, TRAIL, DNA methyltransferase 1, 3a, and 3b, and histone deacetylase inhibitors 1, 2, and 3* in SK-Hep 1, SW620, and PaCa-44 cells treated with TSA at 24h. Quantitative reverse transcription-polymerase chain reaction analysis demonstrated that this compound up-regulated the expression of *Bax, Bak, Bim, DR4, DR5, FAS, FAS-L, TRAIL*, and down-regulated the expression of *Bcl-2, Mcl-1, Bcl-xL, DNA methyltransferase 1, 3a, and 3b* significantly. Asterisks indicate significant differences between treated cells and the control groups. Data are presented as means ± standard error of the mean

The effect of TSA on gene expression was evaluated by quantitative real-time RT-PCR analysis. The result indicated that TSA up-regulated the expression of *Bax, Bak, Bim, DR4, DR5, FAS, FAS-L, and TRAIL *genes and down-regulated the expression of *Bcl-2, Mcl-1, Bcl-xL, histone deacetylase 1, 2, and 3 *in SK-Hep 1, SW620, and PaCa-44 cell lines significantly (Figure 6).

## Discussion

The term “Apoptosis” derives from the Greek language and means trees shedding their leaves, the leaves “falling off” from trees, in autumn. Apoptosis is crucial for physiological processes, embryonic development, tissue homeostasis, and pathological diseases, such as cancer. Normally, it is triggered by two different molecular pathways comprising the death receptor (extrinsic) and mitochondrial (intrinsic) pathways, both of which converge on the activation of the cascade of caspases (27). HDACIs induce apoptosis through the activation of both pathways, intrinsic and extrinsic. They activate the extrinsic pathway via the upregulation of death receptors expression, reduction in c-FLIP, and upregulation of ligands such as TRAIL. Further, they activate the intrinsic pathway through the upregulation of several Bcl-2 family genes such as Bid, Bim, and Bmf (28). Similar to HDACIs, DNA demethylating drugs cause apoptosis via both extrinsic and intrinsic pathways (29). 

The results of current studies indicated that DNA methyltransferase inhibitor zebularine can induce apoptosis in hepatocellular carcinoma SK-Hep 1, human colorectal cancer SW620, and human PaCa-44 pancreatic cancer cell lines. It up-regulated the expression of the genes of the extrinsic apoptotic pathway and pro-apoptotic genes (as mentioned in the result part) and down-regulated the expression of the anti-apoptotic genes, *DNMTs (1, 3a, and 3b)*, *HDACs (1, 2, and 3)* in SK-Hep 1 cell line significantly. Additionally, zebularine up-regulated the expression of the genes of the extrinsic apoptotic pathway and pro-apoptotic genes (as mentioned in the result part) and down-regulated the expression of DNMTs (1, 3a, and 3b), HDACs (1, 2, and 3) in SW620, and PaCa-44 cell lines significantly. We did not observe significant changes in the expression level of *Bcl-2, Mcl-1, and Bcl-xL* in SW620, and PaCa-44 cell lines treated with zebularine. Similarly, in-vitro studies have demonstrated that DNA demethylating agents zebularine and decitabine activate the mitochondrial apoptotic pathway in T cells (30). Similar to our findings, it has been reported that zebularine treatment induces cell-cycle arrest, and apoptosis in the HCC HepG2 cell line through the intrinsic pathway (31). It has been reported that DNA methyltransferase inhibitor 5-Aza-CdR down-regulated MCL-1 leads to apoptotic induction in AML cells (32). In recent studies, it was demonstrated that zebularine and 5-aza-dC down-regulate *DNMT-1, DNMT-3a, and DNMT-3b *in ovarian cancer cell lines (33). Several works have demonstrated that 5-Aza-CdR treatment decrease *DNMT1 and DNMT3a* mRNA expression resulting in a down-regulation of Bcl2 protein in colon cancer cell line HCT-116 (34). Experimental studies have shown that compounds with DNMTI activity such as 5-Aza-CdR generally induce tumor cell apoptosis by DNA demethylation in human cell lines A549, HepG2, Hep3B, A-498, and HCT-116 (35).

In the present work, we reported that histone deacetylase inhibitor TSA can induce apoptosis through both mitochondrial/intrinsic and cytoplasmic/extrinsic apoptotic pathways, in hepatocellular carcinoma SK-Hep 1, human colorectal cancer SW620, and human PaCa-44 pancreatic cancer cell lines through the activation of both extrinsic and intrinsic pathways, it up-regulated the expression of *Bax, Bak, Bim, DR4, DR5, FAS, FAS-L, and TRAIL* genes and down-regulated the expression of *Bcl-2, Mcl-1, Bcl-xL, DNA methyltransferase 1, 3a, and 3b, histone deacetylase 1, 2, and 3* in all three cell lines significantly. Inconsistent with our result, other researchers have indicated that histone deacetylase inhibitor sodium butyrate decreases Bcl-XL protein levels by down-regulating its RNA expression in mesothelioma cell lines. Besides, depsipeptide, as an HDACI, decreases the expression of *Bcl-2, Bcl-XL, and Mcl-1* in multiple myeloma cells (36). In human lymphatic endothelial cells (LEC), TSA-induced apoptosis by cytochrome c release contributed to activating caspases-3, caspases −7, and caspases −9 and the anti-apoptotic proteins down-regulation accompanied by up-regulation of *p21, p27, and p53* (37). Resent in-vitro works have shown that TSA increases the ratio between the levels of expression of anti-apoptotic (*BCL-w and BCL-xl*) and pro-apoptotic (BIM)genes in pancreatic cancer cell lines CFPAC1, Miapaca2, HPAF, PSN1, Panc1 PC, Paca44, RT45P1, and T3M4 (38). Similar to our findings, other researchers have shown that HDACIs play their apoptotic roles through the extrinsic apoptotic pathway. It has been indicated that several HDACIs like SAHA, TSA, m-carboxycinnamic acid bishydroxamide (CBHA), LAQ824 (a cinnamic acid hydroxamate), and MS-275 can up-regulate the expression of *TRIAL-R1 and TRIAL-R2* (39). In pancreatic cancer cell lines Panc1 and MiaPaCa2, treatment with TSA increase the expression of the TRAIL receptor 1 (DR5) (40). Additionally, HDACIs can induce apoptosis through activation of the death receptor pathway (TRAIL and Fas signaling pathways), the up-regulation of TRAIL, DR5, Fas, Fas-L in leukemic blasts (41). 

Meanwhile, the extrinsic and intrinsic pathways are not the only molecular mechanisms of zebularine and TSA. The results of current studies indicated that DNA methyltransferase inhibitor zebularine and histone deacetylase inhibitor TSA can induce apoptosis through both mitochondrial/intrinsic and cytoplasmic/extrinsic apoptotic pathways, in hepatocellular carcinoma SK-Hep 1, human colorectal cancer SW620, and human PaCa-44 pancreatic cancer cell lines. Our finding demonstrated that zebularine induced significant apoptosis in all three cell lines. It up-regulated the expression of *Bax, Bak, Bim, DR4, DR5, FAS, FAS-L, and TRAIL* genes and down-regulated the expression of *Bcl-2, Mcl-1, Bcl-xL, DNA methyltransferase 1, 3a, and 3b, histone deacetylase inhibitors 1, 2, and 3* in SK-Hep 1 cell line significantly. Additionally, zebularine up-regulated the expression of *Bax, Bak, Bim, DR4, DR5, FAS, FAS-L, and TRAIL* genes and down-regulated the expression of *DNA methyltransferase 1, 3a, and 3b* in SW620, and PaCa-44 cell lines significantly. It had no significant effect on *Bcl-2, Mcl-1, and Bcl-xL* gene expression in SW620, and PaCa-44 cell lines. Similarly, in-vitro studies have demonstrated that DNA demethylating agents zebularine and decitabine activate the mitochondrial apoptotic pathway in T cells (30). Similar to our findings, it has been reported that zebularine treatment induces cell-cycle arrest, and apoptosis in the HCC HepG2 cell line through the intrinsic pathway (31). It has been reported that DNA methyltransferase inhibitor 5-Aza-CdR down-regulated MCL-1 leads to apoptotic induction in AML cells (32). In recent studies, it was demonstrated that zebularine and 5-aza-dC down-regulate *DNMT-1, DNMT-3a, and DNMT-3b* in ovarian cancer cell lines (33). Several works have demonstrated that 5-Aza-CdR treatment decrease *DNMT1 and DNMT3a* mRNA expression resulting in a down-regulation of Bcl2 protein in colon cancer cell line HCT-116 (34). Experimental studies have shown that compounds with DNMTI activity such as 5-Aza-CdR generally induce tumor cell apoptosis by DNA demethylation in human cell lines A549, HepG2, Hep3B, A-498, and HCT-116 (35). As mentioned in the result section, TSA induced significant apoptosis in all three cell lines, SK-Hep 1, SW620, and PaCa-44. It up-regulated the expression of *Bak, Bax, Bim, DR4, DR5, FAS, FAS-L, and TRAIL* genes and down-regulated the expression of *Bcl-2, Bcl-xL, Mcl-1, histone deacetylase inhibitors 1, 2, and 3* in these cell lines significantly. Inconsistent with our result, other researchers have indicated that histone deacetylase inhibitor sodium butyrate decreases Bcl-XL protein levels by down-regulating its RNA expression in mesothelioma cell lines. Besides, depsipeptide, as an HDACI, decreases the expression of *Bcl-2, Bcl-XL, and Mcl-1* in multiple myeloma cells (36). In human lymphatic endothelial cells (LEC), TSA-induced apoptosis by cytochrome c release contributed to activating caspases-3, caspases −7, and caspases −9 and the anti-apoptotic proteins down-regulation accompanied by up-regulation of *p21, p27, and p53* (37). Resent in-vitro works have shown that TSA increases the ratio between the levels of expression of anti-apoptotic (*BCL-w and BCL-xl*) and pro-apoptotic (BIM)genes in pancreatic cancer cell lines CFPAC1, Miapaca2, HPAF, PSN1, Panc1 PC, Paca44, RT45P1, and T3M4 (38). Similar to our findings, other researchers have shown that HDACIs play their apoptotic roles through the extrinsic apoptotic pathway. It has been indicated that several HDACIs like SAHA, TSA, m-carboxycinnamic acid bishydroxamide (CBHA), LAQ824 (a cinnamic acid hydroxamate), and MS-275 can up-regulate the expression of TRIAL-R1 and TRIAL-R2 (39). In pancreatic cancer cell lines Panc1 and MiaPaCa2, treatment with TSA increase the expression of the TRAIL receptor 1 (DR5) (40). Additionally, HDACIs can induce apoptosis through activation of the death receptor pathway (TRAIL and Fas signaling pathways), the up-regulation of TRAIL, DR5, Fas, Fas-L in leukemic blasts (41). Meanwhile, the extrinsic and intrinsic pathways are not the only molecular mechanisms of zebularine and TSA. Our previous work indicated that zebularine and TSA can inhibit DNMTs (*DNMT1, DNMT3a, and DNMT3b*), Class I HDACs (*HDACs 1, 2, 3*), and Class II HDACs (*HDACs 4, 5, 6*) and up-regulate CIP/KIP Family (*p21Cip1/Waf1/Sdi1, p27Kip1, and p57Kip2*) resulting in cell apoptosis in colon cancer LS 174T, and LS 180 cell lines (42, 43). Finally, zebularine and TSA can play their apoptotic roles through intrinsic and extrinsic pathways. In some cases, zebularine could not change the gene expression significantly. It may induce a significant change in the expression of the mentioned genes with high concentrations at 48 or 72 h. It may be done with further concentrations or durations. Therefore, the evaluation of this compound with high concentrations and more duration (24 and 48h) is recommended. 

**Table 1:** IC50 values

**Cell line**	**Compound**	**Duration/Hour**	**IC50 Value**
SK-Hep 1	Zebularine	24	56.69
SK-Hep 1	TSA	24	2.439
SW620	Zebularine	24	58.55
SW620	TSA	24	2.454
PaCa-44	Zebularine	24	61.67
PaCa-44	TSA	24	2.894

**Table 2:** The primer sequences of Bax, Bak, Bim, Bcl-2, Bcl-xL, Mcl-1, DR4, DR5, FAS, FAS-L, TRAIL genes, DNA methyltransferase 1, 3a, and 3b, histone deacetylase inhibitors 1, 2, and 3 qRT-PCR was done. The SK-Hep 1, SW620, and PaCa-44 genes

**Primer**	**Primer sequences (5' to 3')**	**Product length**	**Reference**
Bax Forward Reverse	AGTAACATGGAGCTGCAGAGGAT GCTGCCACTCGGAAAAAGAC	77 bp	
Bak Forward Reverse	CCTGCCCTCTGCTTCTGA CTGCTGATGGCGGTAAAAA	82 bp	
Bim Forward Reverse	ATTACCAAGCAGCCGAAGAC TCCGCAAAGAACCTGTCAAT	101 bp	
Bcl-2 Forward Reverse	TGGCCAGGGTCAGAGTTAAA TGGCCTCTCTTGCGGAGTA	147 bp	
Bcl-xL Forward Reverse	TCCTTGTCTACGCTTTCCACG GGTCGCATTGTGGCCTTT	62 bp	
Mcl-1 Forward Reverse	AAAGCCTGTCTGCCAAAT CCTATAAACCCACCACTC	198 bp	
DR4 Forward Reverse	CAGAACATCCTGGAGCCTGTAAC ATGTCCATTGCCTGATTCTTTGTG	299 bp	
DR5 Forward Reverse	TGCAGCCGTAGTCTTGATTG GCACCAAG TCTGCAAAGTCA	389 bp	
FAS Forward Reverse	TTCTGCCATAAGCCCTGTCC TGTACTCCTTCCCTTCTTGG	103 bp	
FAS-L Forward Reverse	GCCTGTGTCTCCTTGTGATG TGGACTTGCCTGTTAAATGGG	222 bp	
TRAIL Forward Reverse	GAAGCAACACATTGTCTTCTCCAA TTGCTCAGGAATGAATGCCC	103 bp	
DNMT1 Forward Reverse	GAGGAAGCTGCTAAGGACTAGTTC ACTCCACAATTTGATCACTAA ATC	206 bp	
DNMT 3a Forward Reverse	GGAGGCTGAGAAGAAAGCCAAGGT TTTGCCGTCTCCGAACCACATGAC	370 bp	
DNMT 3b Forward Reverse	TACACAGACGTGTCCAACATGGGC GGATGCCTTCAGGAATCACACCTC	195 bp	
HDAC1 Forward Reverse	AACCTGCCTATGCTGATGCT CAGGCAATTCGTTTGTCAGA	374 bp	
HDAC2 Forward Reverse	GGGAATACTTTCCTGGCACA ACGGATTGTGTAGCCACCTC	314 bp	
HDAC3 Forward Reverse	TGGCTTCTGCTATGTCAACG GCACGTGGGTTGGTAGAAGT	328 bp	
GAPDH Forward Reverse	TGTTGCCATCAATGACCCCTT CTCCACGACGTACTCAGCG	148 bp	

**Table 3:** Comparative analysis of the effect of zebularine and TSA on SK-Hep 1, SW620, and PaCa-44 cells. Maximal and minimal percentage of apoptosis was seen in SK-Hep 1 cell treated with TSA and PaCa-44 cell treated with zebularine after 24 h of treatment

**Drug**	**Cell line**	**Dose/μM**	**Duration/h**	**Apoptosis (%)**	***P*** **-value**
Zebularine	SK-Hep 1	56.69	24	96.73	*P* < 0.001
Zebularine	SW620	58.55	24	76.6	*P* < 0.001
Zebularine	PaCa-44	61.67	24	63.1	*P* < 0.001
TSA	SK-Hep 1	2.439	24	97.99	*P* < 0.001
TSA	SW620	2.454	24	81.34	*P* < 0.001
TSA	PaCa-44	2.894	24	85.74	*P* < 0.001

## Conclusion

In conclusion, our results indicated that histone deacetylase inhibitor TSA can induce its apoptotic effects through both mitochondrial/intrinsic and cytoplasmic/extrinsic apoptotic pathways in hepatocellular carcinoma SK-Hep 1, human colorectal cancer SW620, and human PaCa-44 pancreatic cancer cell lines. Further, zebularine can induce apoptosis by an extrinsic apoptotic pathway in three cell lines. It had no significant effect on *Bcl-2, Mcl-1, and Bcl-xL* gene expression in SW620, and PaCa-44 cell lines. Therefore, TSA can induce a stronger apoptotic effect through the activation of both pathways. Finally, both agents induced apoptosis significantly, which provided the theoretical basis for the clinical application of these compounds.
